# Audiological and vestibular evaluations in vitiligo patients

**DOI:** 10.17305/bjbms.2021.5703

**Published:** 2021-08-09

**Authors:** Alessandra Manno, Annalisa Pace, Giannicola Iannella, Valeria Rossetti, Roberta Polimeni, Alessandro Milani, Salvatore Cocuzza, Antonino Maniaci, Giuseppe Magliulo

**Affiliations:** 1 Department of Organi di Senso, Sapienza University, Rome, Italy; 2 Department of Otolaryngology, University of Catania, Catania, Italy

**Keywords:** VEMPs, vitiligo, vestibular system, hearing loss, otic melanocytes

## Abstract

The aim of this paper was to investigate audiological abnormalities and potential vestibular injury in a sample of vitiligo subjects. Thirty-five patients with non-segmental vitiligo (NSV) were enrolled in the study. They underwent pure tonal audiometry (PTA), vestibular Fitzgerald-Hallpike caloric test, C-VEM, and ocular VEMP (O-VEMP) testing. The χ^2^ test and multiple regression analysis were performed. At PTA, 69% of patients presented with bilateral hearing loss, 8% monaural hearing loss, and 23% normal values. Bilateral caloric stimulations were performed and demonstrated that 14% of patients had a monolateral and 9% had a bilateral pathological response. VEMPs analysis showed that 20% of patients had no O-VEMPs response and 3% had no cervical VEMPS (C-VEMPs) response. Comparison between the normal values of healthy subjects and NSV patients showed an alteration of VEMPs in 44%. Multiple regressions showed no statistical differences. We propose a specific diagnostic protocol employing PTA, bithermal caloric tests, C-VEMP, and O-VEMP testing to evaluate audio-vestibular damage. Our data were concordant with the anatomic-physiological melanocytic distribution and their possible degeneration linked with NSV.

## INTRODUCTION

Vitiligo is an acquired, sometimes familiar, depigmentary disease resulting from selective destruction of melanocytes with characteristic pearl-white skin patches of different shapes and sizes [1]. It is clinically classified as either segmental vitiligo (SV) or non-SV (NSV). Since many studies have demonstrated that melanocytes are localized in different districts of the inner ear, contributing to labyrinth fluid homeostasis, some authors investigated the association between vitiligo and hearing loss [1] considering it as a potential pathogenic factor, damage of the otic melanocytes. However, even though the posterior labyrinth also presents melanin components, only two studies attempted to investigate the problem [2,3]. Furthermore, the latter only partially considered a possible deficit of the posterior labyrinth, since only Cervical Vestibular Evoked Myogenic Potentials or videonystagmography were performed.

The VEMPs (ocular VEMPs [O-VEMPs] and cervical VEMPs [C-VEMPs]) are vestibular exams able to evaluate the otolith organs, thus investigating the utricular and saccular functions and differentiating, indirectly, superior, and inferior nerve involvement. Hence, it may be useful to observe indirect and latent deficits of the vestibular system in asymptomatic patients too.

The aims of this paper were to investigate audiological abnormalities and the possibility of vestibular damage in a group of vitiligo patients using a diagnostic protocol including pure tone audiometry, caloric test, C-VEMPs, and O-VEMPs as well as to evaluate, in the vestibular diagnostic protocol, the implementation of traditional types of vestibular examinations with new methods for obtaining a more precise evaluation.

## MATERIALS AND METHODS

The diagnosis of vitiligo was performed by dermatologists. All patients had a definitive diagnosis of vitiligo, a thorough recording of medical history, clinical examination, and an accurate evaluation of lesions made using a Wood’s lamp. To ensure a homogeneous sample, only patients with NSV were enrolled.

Patient characteristics, pharmacological treatment, hearing loss, episodes of imbalance, and or dizziness were investigated at the time of evaluation.

After otoscopic examination, participants underwent pure tone audiometry at the frequencies of 0.25, 0.5, 1.0, 2.0, 4.0, and 8.0 kHz for air conduction and between 0.25 and 4.0 kHz for bone conduction. The American Speech-language - Hearing Association classification was used to evaluate the auditory performance of vitiligo patients (Table 1) [4,5]. To carefully investigate any possible vestibular involvement, all patients underwent the Fitzgerald-Hallpike caloric vestibular test, C-VEMPs, and O-VEMPs testing.

**TABLE 1:**
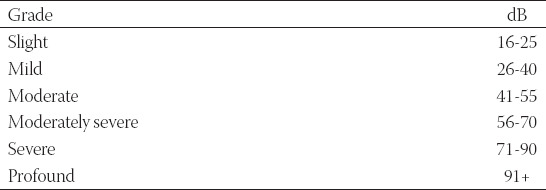
American Speech-language-Hearing Association classification

The Fitzgerald- Hallpike caloric vestibular test calculates latency prior to the appearance of spontaneous nystagmus following provocative cold (30°C) and warm stimuli (44°C) (normal value, canal paresis of less than 15%).

C-VEMPs are performed with MK22 Amplaid that uses an air-conducted auditory stimulus to evoke the reflex with the patient lying down. Five electrodes are located on the patient: The two reference ones are on sternocleidomastoid muscles (SCM); the two exploring ones are over the middle of the clavicles; and the ground one is on the forehead. The patient is then invited to raise his head, curving his neck for the entire duration of the auditory stimulus. In this way, a bilateral simultaneous contraction of the SCMs muscles is obtained.

Before each registration, electrode impedance was fixed below 5 kΩ, while the stimulus was amplified and passband-filtered from 15 to 2000 Hz. The auditory stimulus has an intensity of 130 dB SPL at a rate of 4/s. The binaural acoustic stimulus was based on 500 Hz logon of negative polarity. Logon consisted of negative semi-waves equivalent to the rarefaction phase of an acoustic stimulus.

THD 49 earphones were used during each evaluation, providing two hundred stimuli. Each registration was reproduced twice to obtain a reliable response and an average of the results was computed for the first positive-negative peak of the VEMPs [5].

Various parameters to assess any impairment of the C-VEMPs and O-VEMPs were investigated. The reference group consisted of 100 healthy age-matched volunteers (average age 42.5 years; range 25-79 years) who had no vitiligo, no vertigo or nystagmus, and normal neurologic evaluation. Tests were considered abnormal if they were outside the average value ±2SDs.

C-VEMPs evaluation was considered pathological when there was no response or in the event of a decreased amplitude of the p13-n23 complex.

The normal values are described as mean ± 2SDs: p13 latency was 16.25 ± 1.52 ms; n23 latency was 25.4 ± 2.8 ms; and p13-n23 amplitude was 39.75 ± 21.68 μV.

O-VEMPs are calculated by a hand-held mini-shaker (4810, Bruel and Kjaer; Naerum, The Denmark) that produces a bone-conducted vibration. The patient is placed in a supine position and is located about 15~30° backward at a distance of 2 m from a visual target.

Five electrodes are positioned: Two exploring ones below the center of the lower eyelids; two reference electrodes 1-2 cm below the exploring ones and the ground one on the forehead. Recorded stimuli were amplified and band-pass filtered from 20 to 500 [5].

Impairment of O-VEMPs was defined if there was a lack of n10 waves. Moreover, latency and amplitude of n10 were compared with our age-related normative reference range (15.20 ± 2DS): An increased value of latency or decreased value of amplitude was considered pathological. The abnormal values were expressed as mean ± 2SDs: n10 wave latency was 10.29 ± 0.6 ms; N10 wave amplitude was 6.57 ± 2.01 μV. C-VEMPs were estimated ipsilaterally to the area of the stimulation, while O-VEMPs were performed on opposite sides owing to their crossed vestibulo-ocular responses.

### Ethical statement

Sapienza University Ethical Committee approved this study (RIF: CE4324) in accordance with the Helsinki Declaration. Informed consent was signed by each patient.

### Statistical analysis

A descriptive analysis of data was performed, expressing results as counts (percentage) for groups of patients. The χ^2^ test and multiple regression analysis were considered for the comparative relation of the different groups. The *p*-value obtained was considered significant for a value <0.05. The statistical software used was the R Project for Statistical Computing version 4.0.

## RESULTS

Thirty-five patients suffering from NSV (Caucasian race, mean age 39.26 years, range 21-69 years; 15 women and 20 men) were investigated (nine patients with a mucosal manifestation and 26 patients with the acrofacial form).

One patient (3%) was taking systemic corticosteroids at the time of the evaluation, 6 (17%) patients were in treatment with phototherapy alone, 22 (63%) patients were being treated with local application of cream (alone or in combination with supplements or phototherapy), and only 6 (17%) of them were not undergoing either pharmacological or phototherapy (Table 2). Hearing loss symptomatology was referred by 22 patients (63%) and five patients (14%) referred to imbalance episodes while bouts of dizziness were reported by just 1 (3%) patient.

**TABLE 2:**
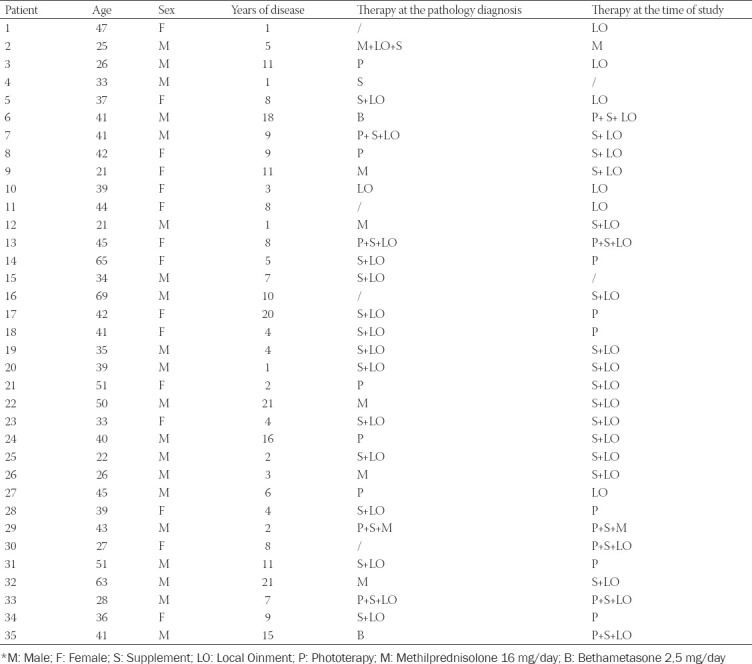
Patient characteristics, clinical conditions, and pharmacological treatment

Pure tone audiometry showed that 24 (69%) patients were suffering from bilateral hearing loss, 3 patients (8%) had a monaural hearing loss, and 8 (23%) patients presented a normal exam.

Bilateral caloric stimulations produced a unilateral pathological response in 5 patients (14%), while 3 patients (9 %) had a bilateral pathological response (Table 3).

**TABLE 3:**
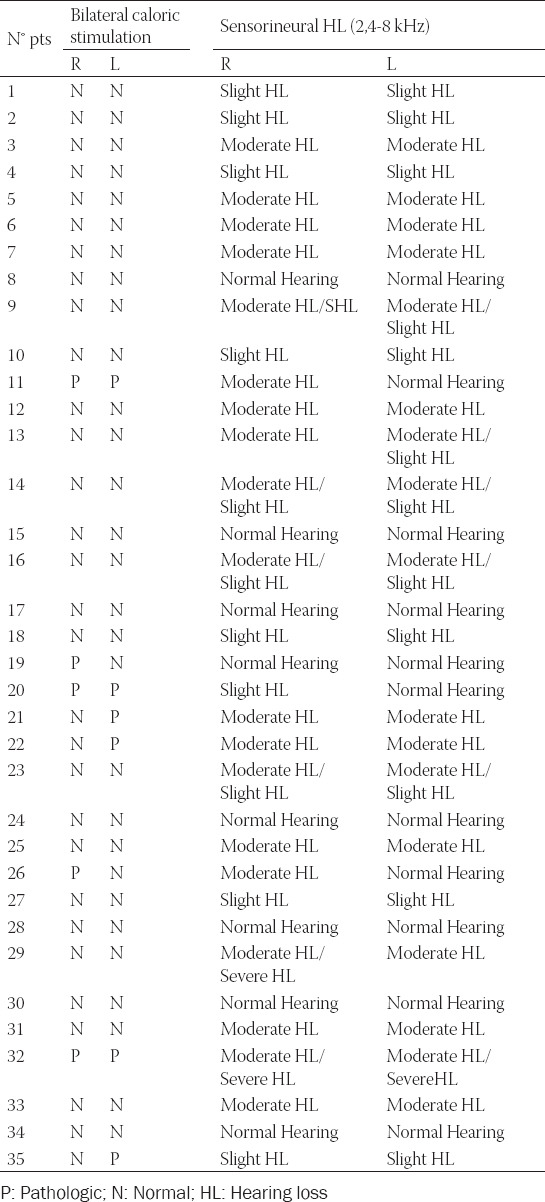
Value resulted from pure tone audiometry and caloric test

The results of VEMPs were analyzed in two different ways, owing to the lack of standardized results: First, the percentage of patients with absent responses was estimated and then the percentage of patients with anomalous values with respect to the healthy subjects tested. At the first analysis, 7/35 (20%) patients had no O-VEMPs response and 1/35 (3%) had no C-VEMPs response. Only two of these patients had a simultaneous alteration at caloric stimulation. Comparison between the normal values of healthy subjects and NSV patients showed alteration of VEMPs in 14/35 patients (40%): 4/14 (29%) presented an anomalous value of both C-VEMPs and O-VEMPs, while 9/14 (64%) had a modification of O-VEMPs alone and just 1/14 (7%) had an alteration of C-VEMPs alone.

O-VEMPs revealed an altered latency of N10 in 11 patients: Six patients presented an absent response bilaterally and 1 patient unilaterally; 1 patient had an increased latency unilaterally and 3 bilaterally. Moreover, 2 patients had a reduced value of amplitude unilaterally (Table 4).

**TABLE 4:**
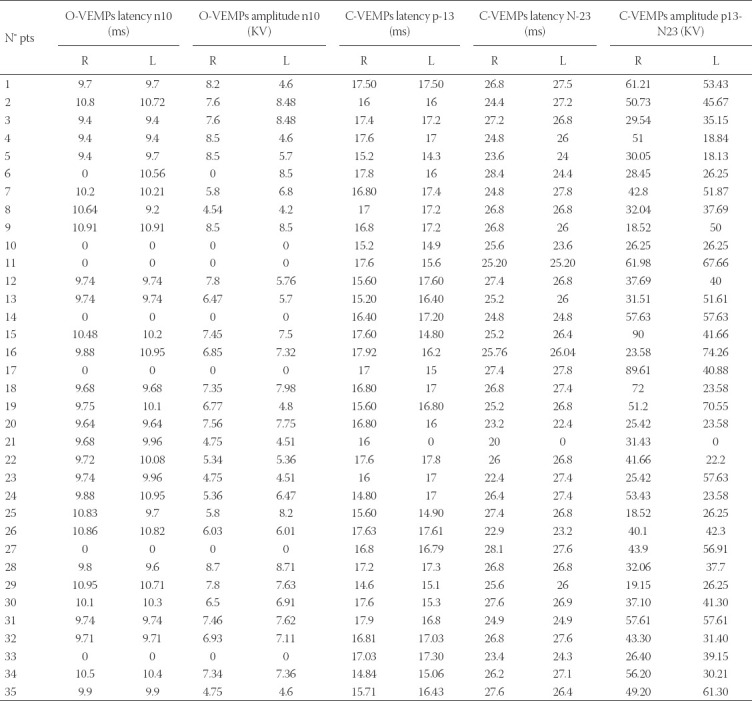
Value resulted from C-VEMPs and O-VEMPs

C-VEMPs showed an altered latency of p13 in 5 patients: One of these presented an absent response unilaterally while the other four had a reduced latency unilaterally. Latency of n23 was altered in only 2 patients, 1 with an absent response and 1 with a reduction, both mononolaterally. Finally, the amplitude of the p13-n23 complex was unilaterally absent in only 1 patient (Table 4).

Multiple regression was used to evaluate the relationship between age and O-VEMP N10 latency, as well as age and O-VEMP N10 amplitude in both ears, showed no statistical differences (*p* = 0.26 and *p* = 0.19, respectively). Similar findings were observed using multiple regression between age and C-VEMP p13 latency and age andN13 C-VEMPs amplitude in both ears (*p* = 0.37 and *p* = 0.36, respectively). Finally, multiple regression for the relationship between age and C-VEMP p13-n23 amplitude in both ears did not show any statistical differences (*p* = 0.33).

## DISCUSSION

Vitiligo is a common, chronic, depigmenting skin disease constituted by the destruction of melanocytes (epidermis, the mucous membranes, eyes, and in some hair bulbs) [6]. Moreover, involvement of leptomeninges, retinal pigment epithelium, uveal tract, and inner ear melanocytes is reported.

Typically, the destruction of melanocytes in the skin produces hypo-pigmented and non-symptomatic macules with clearly defined and demarcated margins. An association between vitiligo and ocular, hearing, and autoimmune pathologies is reported in the literature [7,8]. The higher percentage also includes very mild SNHL as described by Fleissig et al. Audiological abnormalities may be observed in other hypopigmentation disorders (albinism, Waardenburg’s syndrome, Vogt-Koyanagi-Harada syndrome, and Alzendrini syndrome) [9].

Cochlear stria vascularis is anatomically composed of melanin-producing intermediate cells (IC) that play a critical role in endocochlear potential (EP). Melanin is an antioxidant and metal chelator that protects the stria and organ of Corti against pathogenic agents (noise and ototoxicity). In animal trials, it was demonstrated that toxin ablation of IC-induced deafness [10]. Moreover, the lack of melanin in Albino mice produced an increased age-related EP decline with consequent hearing loss.

However, there are discrepancies in the literature regarding the specific influence of vitiligo on the auditory threshold, recently a larger number of controlled studies have confirmed that SNHL is significantly more common among vitiligo patients, even though most of them are clinically asymptomatic for audiometric abnormalities [11].

In 2019, Rahimi et al. [12] performed a case–control evaluating distortion product otoacoustic emissions (DPOAE) and pure tone audiometry (PTA) without any statistically significant differences between groups. This was in line with other authors [13] but, in contrast with those who reported a higher incidence of SNHL in patients with vitiligo, ranging from 4 to 68.8%, compared to healthy controls [14-19].

In our series, 24 (69%) patients were found to suffer from bilateral hearing loss and 3 (8%) patients presented a monaural hearing loss, in particular at the high-frequency range, whereas 8 (23%) had normal hearing levels. The incidence of hearing loss at the range of high frequencies is concordant with other papers that evidenced significantly higher thresholds among vitiligo patients at frequencies between 4 and 8 kHz.

Fleissig et al. evaluated 16 patients affected by vitiligo and 16 healthy controls without any history of noise-induced hearing loss. The shapes of the audiograms [9] revealed a significant common “notch shape” at the higher frequencies.

However, Moghaddam et al., in their recent cross-sectional study, did not find any relationship between the severity of skin involvement and that of hearing loss in vitiligo patients [20]. In addition, they found no relationship between conductive and sensorineural hearing losses, the incidence and duration of clinical symptoms, or the extent of skin involvement in vitiligo patients.

A possible explanation of high-frequency range damage is related to the different localization of melanin in the cochlea. Studies carried out on mice and human temporal bone have reported that its distribution is lower in the basal than in apical turn in the Rosenthal canal. Therefore, since the basal turn is the portion that analyzes the high frequencies, a lack of melanin in vitiligo patients may produce an early deterioration of these frequencies [21].

Other authors suggest that the pathogenetic mechanisms of vitiligo could be attributable to a systemic event since vitiligo is associated with ocular and auditory abnormalities including SNHL [8].

At present, in the literature, only two studies have reported evidence regarding vestibular function in patients affected by vitiligo and only one underwent VEMPs analysis.

Melanocytes of the vestibular system can mainly be found in the dark cell area of the utricle and the ampulla of the semicircular canals [22], closely aligned with the dark cells. In fact, it is uncommon to find them in the saccule and the other semicircular canals, because of the absence of dark cell epithelium in these locations.

Subepithelial melanocytes and the dark cell epithelium have a similar function to the marginal and IC of the stria vascularis. Therefore, they may be useful for endolymph homeostasis.

Moreover, in the literature, it is claimed that melanin, activated by acoustic and electrical stimulation, is a protective agent against environmental injury and semi-conductive properties, responding to acoustic and electrical stimulations and may transform energy states into molecular rotation and vibration.

VEMPs testing is a relatively new vestibular exam, able to study the otolith organs, investigate utricular and saccular functions, and differentiate between superior and inferior nerve involvement whereas traditional methods, such as caloric vestibular testing, which are only able to investigate lateral semicircular canal dysfunction [23-25]. The utricular nerve and superior and lateral ampullary ones connect together to form the superior vestibular nerve. The same occurs for the inferior vestibular nerve that is created by the union of the saccular nerve and posterior ampullary one. Therefore, VEMPs testing has been used to study many vestibular pathologies such as vestibular neuritis, superior canal dehiscence syndrome, vestibular migraine, and Meniere’s syndrome [5, 26-30] since it studies otolithic function in both its saccular (C-VEMPs) and utricular (O-VEMPs) system [28].

Mahdi et al. performed C-VEMPs to detect vestibular involvement in 21 patients with vitiligo. Pathologic values of C-VEMPs were found in 6 patients: An absent response (4.76%) and a prolongation of latency were observed in 5 patients (23.80%). These results are incomplete because O-VEMPs and Fitzgerald-Hallpike caloric vestibular stimulation were not included, despite the prevalence of melanocytes in the utricle and the ampullae of the semicircular canals.

Dawound, on the other hand, evaluated vestibular ­function performing videonystagmography and found a vestibular disorder in 50% of their patients but this test is operator-dependent and only partially objective for the vestibular phenomenon.

Therefore, accurate analysis of the vestibular system should include both traditional caloric testing and the more recent vestibular tests such as C-VEMPs and O-VEMPs.

Our data were in agreement with the anatomical-physiological distribution of melanocytes and their possible degeneration linked with NSV. The highest incidence of altered results was, in fact, observed by Fitzgerald-Hallpike caloric vestibular stimulation and O-VEMPs.

Although the statistical analyses did not provide significant results, it should be noted that the use of caloric vestibular stimulation identified anomalies in 8/35 patients (23%), of O-VEMPs in 13/35 patients (37%) and C-VEMPs in only 5/35 (15%).

To avoid a possible over-estimation of the findings observed, due to the lack of standardized results for the VEMPs equipment, the number of pathological results was also estimated, calculating only absent responses to VEMPs. Results reported absent VEMPs in 8 patients (7 with O-VEMPs and 1 with C-VEMPs) similar to the 8 patients identified by caloric stimulation. However, it is important to bear in mind that only 2 patients with an absent VEMPs response had a simultaneous alteration at caloric stimulation.

These results demonstrate that the above techniques used alone are inadequate for performing a vestibular diagnosis.

Our results showed a predominance of hearing loss rather than vestibular deficit. A hypothesis is based on the ability of the vestibular system to perform a central compensation that allows patients to be asymptomatic even when there is a latent vestibular deficit. However, it needs to be proved by performing electrophysiological evaluation in addition to auditory and vestibular ones.

The association of video head impulse testing (v-HIT) and VEMPs, in fact, is the only method able to test all five vestibular end organs non-invasively. Therefore, VEMPs, associated with other vestibular tests, may indicate a possible vestibular disorder.

The same should be considered for the auditory system that should be completely evaluated by ABR and DPOE. This could be considered a limitation of the study: However, a case-control protocol that also includes v-HIT, ABR, and DPOE in patients affected by vitiligo is currently underway.

## CONCLUSION

Many studies have confirmed that melanocytes are present in the inner ear. In the vestibular labyrinth, melanocytes are present in the utricle, saccule, pars commune, ampulla, endolymphatic duct, and sac. We propose a specific diagnostic protocol employing bithermal caloric testing, C-VEMPs, and O-VEMPs to prove or exclude vestibular damage even in asymptomatic patients and to obtain a more precise definition of the vestibular site involved. A larger sample study with a protocol including v-HIT, to detect the distribution of semi-circular involvement and to better identify possible selective damage of the vestibular nerve is needed for a complete evaluation of the vestibular system in these patients.
